# 

**Published:** 1903-09-26

**Authors:** 


### NOTICE TO CORRESPONDENTS.

All MSS., Letters, Books for Review, and other matters intended for the Editor should be addressed The Editor, "The Hospital," The Hospital Buildings, 28 & 29 Southampton Street, Strand, W.C.

The Editor cannot undertake to return rejected MS., even when accompanied by stamped directed envelope.

**Notice to Advertisers and Subscribers.**

All Advertisements, Orders for Copies of The Hospital, and Business Communications should be addressed to **THE MANAGER, (Not to the Editor)**, The Hospital, 28 & 29 Southampton Street, Strand, London, W.C.

The Cost of Subscription, post free, is as follows.—Per week, 3½d.; per quarter, 3s. 9d.; per half-year, 7s. 6d.; per annum, 15s. Foreign and Colonial:—Per annum, 19s. 6d., payable, in all cases, in advance.

**NOTICE.—Vol. XXXIII. of "The Hospital" (October 1902 to March 1903) in handsome cloth binding, will shortly be on sale at the office of this Journal, price 7s. 6d. Cases for binding the Numbers contained in this Volume 1s. 6d. Index to Vol. XXXIII., 2½d., post free.**

**The monthly part for October will be ready on October 1. Price 1s., by post 1s. 3d.**

### BOOKS RECEIVED.

Baillière, Tindall and Cox.

"Practical Details of Cataract Extraction." By H. Herbert.

Harrison and Son.
"England's Future." By W. Culver James, M.D.

Hodder and Stoughton.
"Story of My Life." By Helen Keller.

J. Wright and Co., Bristol.

"Membranous Catarrh of the Intestines, Nephritis, Obesity." By Von Noorden.

### Scraps and Gleanings.

There are 64,110 paupers and imbeciles of all ages in London wholly dependent upon public support.

The White Star Liner *Britannic*, which has sailed over 2,000,000 miles, has had the same engines and boilers in use for 25 years.

Of the 12,575 candidates in the Oxford Local Examinations, the results of which have lately been issued, 9,622 obtained certificates.

In the countries of the Postal Union 50,000.000 letters were undelivered in 1901, and of 26,000,000 of these the senders could not be traced.

A German lady, aged 72, has, it is stated, just accomplished the feat of walking from Montreux to Chamonix, crossing the Col de Balme and Forclaz Pass, a d stance of about 50 miles in 13 hours.

The Church of England Temperance Society has decided to start a "testing home" for drunkards who have been confined in an inebriate home as an intermediate stage before they return to the world.

The Countess of Warwick recently visited the West Ham and East London Hospital at Stratford, and wrote the following in the Visitors' Book:—"Visited all the Hospital, and found a bright spot in West Ham needing the support of all who work in the cause of humanity—the first example being the devotion of the medical staff and the nurses." The Hospital authorities are still working energetically towards the enlargement of their well-managed institution, and during the autumn, by means of a garden party, meetings, and in other ways, hope to obtain substantial additions to the building fund.

### The Student of Medicine.

### APPOINTMENTS.

**Percy T. Adams**, M.R.C.S.Eng., D.P.H., L.S.A., Deputy Medical Officer of Health of the Orange River Colony. **William Cockburn**, M.B., C.M.Aberd., Certifying Factory Surgeon for the Old Meldrum District of Aberdeenshire. **John Findlay**, M.B., Ch.B., Surgeon and Agent for the Admiralty at the Coastguard Station, Rattray Head, Aberdeenshire. **L. W. Light**, M.R.C.S., L.R.C.P., Medical Officer for the Bradwell District of the Maldon Union. **A. Dingwall Kennedy**, M.B., Ch.B.Glasg., provisionally, Medical Officer to the Ballachulish Quarrymen's Medical Club and Friendly Societies. **Wilfred E. Knight**, M.B.Edin., L.R.C.P.E., Senior House Surgeon to the West Ham and East London Hospital. **F. T. Churchill Linton**, M.B.Edin., Junior House Surgeon at West Ham and East London Hospital, Stratford, E. **R. W. Nesfield**, M.B., Ch.B.Vict., Medical Officer for the Cumberworth District of the Huddersfield Union. **E. Payne**, M.B., M.R.C.S. L.R.C.P.Lond., District Medical Officer of the Dover Union. **A. Robertson**, M.D., D.P.H., Clinical Assistant to the Chelsea Hospital for Women. **A. R. Henchley**, M.D., Clinical Assistant to the Chelsea Hospital for Women. **C. Howard Morgan**, M.R.C.S., L.R.C.P., Clinical Assistant to the Chelsea Hospital for Women.

### "The Hospital" Institutional Library.

"Hospitals and Asylums of the World." 4 Vols., with a Portfolio of Plans, complete £12 12s.

"Burdett's Hospitals and Charities, 1903." 5s. net.

"Burdett's Official Nursing Directory, 1903." 3s. net.

"Cottage Hospitals, General, Fever, and Convalescent." 10s. 6d.

"Uniform System of Accounts for Hospitals and Public Institutions." New Edition. 4s. net.

"Hospital Expenditure: The Commissariat." 2s. 6d.

"A Legal Handbook for the Use of Hospital Authorities." 2s. 6d.

"The Cottage Hospital Case Book or Register of Patients." 10s. and 12s. 6d.

All these are published by the Scientific Press, Ltd., and may be obtained through any bookseller, or direct from the publishers, 28 and 29 Southampton Street, Strand, London, W.C.

**"Valuable little handbook."**
*Nursing Notes.*

**THE BURDETT SERIES.**

**"Can be strongly recommended to nurses."**
*Treatment.*

### THE BURDETT SERIES OF POPULAR TEXT-BOOKS on NURSING.

**Each Volume contains about 100 pages written by a competent authority on the subject treated.**

Small Crown 8vo. Bound in cloth boards. Price **1/-** each post free; or the Series complete **10/-** post free.

No. 1.—**PRACTICAL HINTS ON DISTRICT NURSING.** By Miss Amy Hughes.

No. 2.—**HOSPITAL AND PRIVATE NURSING.** By Miss S. E. Orme.

No. 3.—**THE MIDWIVES' POCKET-BOOK. With Prefatory Chapter on the Midwives' Bill.** By Miss Honnor Morten, Author of "The Nurse's Dictionary," &c., &c.

No. 4.—**FEVERS AND INFECTIOUS DISEASES:** their Nursing and Practical Management. By William Harding, M.D.Ed., M.R.C.P.Lond., Author of "Mental Nursing," &c.

No. 5.—**NOTES ON PHARMACY AND DISPENSING FOR NURSES.** By C. J. S. Thompson.

No. 7.—**THE CARE OF CONSUMPTIVES.** By W. H. Daw, M.R.C.S., L.R.C.P.Lond.

No. 8.—**HOME NURSING OF SICK CHILDREN.** By J. D. E. Mortimer, M.B., F.R.C.S., L.S.A.

No. 9.—**MENTAL NURSING.** By William Harding, M.D.Ed., M.R.C.P.Lond.

No. 10.—**SICK NURSING AT HOME.** By Miss L. G. Moberly.

No. 11.—**GYNÆCOLOGICAL NURSING.** By G. A. Hawkins-Ambler, F.R.C.S., Author of "The Commonwealth of the Body."

No. 12.—**SYLLABUS OF LECTURES TO NURSES.** By Andrew Davidson, M.D.

*To be obtained of all Booksellers, or of*

The SCIENTIFIC PRESS, LTD., 28 & 29 Southampton Street, Strand, London, W.C.

